# Death of Medical Men in Boston

**Published:** 1846-03

**Authors:** 


*Death of Medical Men in Boston.*—With a pretty strong medical corps in proportion to the population, we recollect of but one death of a physician in the city during a period of nearly a year. That individual's decease was chronicled last week. Notwithstanding the harmony of the profession and their admirable social organization, it was remarked that only four of his medical brethren were at the funeral. It should have been otherwise. All other associations pay a more strongly-marked expression of respect to the memory of their departed members.—*Ibid.*

